# S3: An AI-Enabled User Continuous Authentication for Smartphones Based on Sensors, Statistics and Speaker Information

**DOI:** 10.3390/s21113765

**Published:** 2021-05-28

**Authors:** Juan Manuel Espín López, Alberto Huertas Celdrán, Javier G. Marín-Blázquez, Francisco Esquembre, Gregorio Martínez Pérez

**Affiliations:** 1 Department of Information and Communications Engineering (DIIC), University of Murcia, 30100 Murcia, Spain; juanmanuel.espin1@um.es (J.M.E.L.); gregorio@um.es (G.M.P.); 2 Communication Systems Group (CSG), Department of Informatics (IfI), University of Zürich UZH, CH-8050 Zürich, Switzerland; huertas@ifi.uzh.ch; 3 Department of Mathematics, University of Murcia, 30100 Murcia, Spain; fem@um.es

**Keywords:** continuous authentication, smartphone, sensors, applications usage, speaker recognition, artificial intelligence

## Abstract

Continuous authentication systems have been proposed as a promising solution to authenticate users in smartphones in a non-intrusive way. However, current systems have important weaknesses related to the amount of data or time needed to build precise user profiles, together with high rates of false alerts. Voice is a powerful dimension for identifying subjects but its suitability and importance have not been deeply analyzed regarding its inclusion in continuous authentication systems. This work presents the S3 platform, an artificial intelligence-enabled continuous authentication system that combines data from sensors, applications statistics and voice to authenticate users in smartphones. Experiments have tested the relevance of each kind of data, explored different strategies to combine them, and determined how many days of training are needed to obtain good enough profiles. Results showed that voice is much more relevant than sensors and applications statistics when building a precise authenticating system, and the combination of individual models was the best strategy. Finally, the S3 platform reached a good performance with only five days of use available for training the users’ profiles. As an additional contribution, a dataset with 21 volunteers interacting freely with their smartphones for more than sixty days has been created and made available to the community.

## 1. Introduction

In recent years, smartphones have become an essential tool for work, leisure and even access to financial and other personal services. With the development of technology, functionalities offered by smartphones are ever-increasing. Among the most used can be found email, social networking, money transfer and online shopping. All the apps used for these purposes store information and data that are sensitive to the user’s privacy, which, too frequently, becomes accessible once access to the device is obtained.

To protect users’ privacy, developers of applications and operating systems (OS), and even smartphone companies, introduce different authentication systems. These systems offer the owner of the phone the possibility of using a variety of authentication mechanisms, such as patterns, passwords, and even biometrics, tokens, or cards [1]. However, all these techniques only allow for an immediate and application-dependent authentication of the user. This means that, if the user interacts with different apps, or if the authentication for a given application expires, the user may have to repeat the authentication several times. On the contrary, some applications that use sensitive information never request user authentication for the sake of usability, or they just request authentication the first time used in a device. Some examples of this kind of apps and services are WhatsApp, Google, Facebook, and Instagram, where the user’s profile is always open.

To reduce both intrusiveness and the number of authentication requests of traditional authentication systems, smartphones can use continuous authentication systems. Continuous authentication aims at providing identity confirmation by constantly assessing the confidence in the current user’s identity, therefore improving smartphone usability [2]. Continuous authentication validates users continuously, as they interact with the device, with no explicit user intervention. More in detail, these systems analyze users’ behavior on the device using data from various sources, being among the most common: phone sensors, keystrokes, app usage statistics, and even patterns of finger movements on the screen device. After a behavioral profile is created for a given user, new real-time data is assessed with the model to obtain a similarity score. Different algorithms are described in the literature to create such users’ profiles, with Artificial Intelligence (AI) techniques being the most used nowadays.

Despite benefits provided by current solutions, large amounts of data may be required to generate accurate users’ models. In some cases, more than fifteen days of data are reported as necessary for obtaining an acceptable model performance [3], although in too many other published works [4,5] this information is simply not provided. Another problem with continuous authentication systems is that they usually suffer a high percentage of false acceptance, opening a door to illegitimate use. An additional limitation of most systems is that users’ models are linked to the particular device analyzed (device-dependent). When users replace the device, new models have to be generated, instead of reusing previous ones.

To mitigate these problems, many researchers suggest other biometrics as potential sources of additional data that can help in the authentication process. Other relevant biometrics available on current smartphones are fingerprint, facial, and voice data. Among these alternatives, only voice is a communication channel. Voice is the second most frequently used communication channel, second only to text messages, although it has to be pointed out that messages do not include biometric information of the sender. People are remarkably good, in a natural way, to discriminate whether a voice belongs, or not, to a given known person. Humans can even recognize the identity of a small group of acquaintances by their voice. However, the use of voice in smartphones is limited to particular situations: phone calls and voice messages. The irruption of messaging apps, such as WhatsApp or Telegram, has caused a considerable reduction in the number of calls from, or to, a user. Yet, many users communicate using voice messages, as they are easy to record and allow for more natural and meaningful messages, as they can include intonations that cannot be conveyed by a text message. Voice is also used to operate the device in hands-free mode, such as when ordering calls or controlling music while driving. Besides, voice biometrics are already successfully used in other situations, such as authenticating the user on a call to offer a personalized service or detecting fraud in phone calls [6], among others [7].

Voice has been already reported as part of proposals for continuous authentication, although it has not been studied in detail. Shi et al. [5] included the voice in the prototype design, but they did not collect audio samples or performed experimentation. In addition, Crawford et al. [8] included the voice along with the key stroke, but they did not evaluate the system with real data. Feng et al. [4] used voice as part of their continuous authentication system, to detect both the type of activity performed by the user and changes of speaker. These authors used the Android speech recognition API, but provided no further details. In summary, despite the evident potential of voice, its usage in continuous authentication systems has not been studied in detail, and the following open research questions need additional efforts from the research community.

1. Can voice be seamlessly added as another data source to improve the accuracy of continuous authentication systems?
2. When data from two, or more, different sources (voice included) are available, is it better to use the data separately or to group them in some way?
3. How does the amount of data belonging to different dimensions (voice, applications, or sensors), and therefore the time required to create behavioral profiles, affect the precision of authentication solutions?
4. How relevant is voice compared to other data sources used in continuous authentication systems?
5. Are there publicly available datasets combining voice and other dimensions that can be used to evaluate this relevance?

To answer the previous questions, the main contributions of this work are the following:

- The design and implementation of the S3 Platform—Sensors, Statistics, and Speaker Platform—an AI-enabled continuous authentication system for smartphones that considers data from sensors, statistics of applications, and voice. The S3 Platform is composed of: (i) a smartphone application that collects data from the previous three dimensions, and (ii) an intelligent framework in charge of creating user’s behavioral profiles and calculating the authentication level of the subject interacting with the mobile device in real time.
- The creation of the S3 dataset, which models the behavior of twenty-one volunteers interacting with their smartphones for more than sixty days. The S3 platform has been used to monitor and store data from sensors, application usage statistics, and the speaker of each volunteer’s smartphone. The S3 dataset is publicly available in [9].
- The validation of the S3 platform through a set of experiments, using anomaly detection techniques, and the S3 dataset, to authenticate users in a continuous fashion. In particular, the performed experiments have explored the platform precision using the three different kind (In this work, a kind of data would refer to one or more different attributes that share a similar nature. For example, the data attributes provided by different sensors, or data attributes that are statistics of usage, would be two kinds of data. It would also be referred to as “dimension”.) of data (sensors, applications statistics, and voice), both separately and in all possible group of kind combinations. The experiments showed that voice is the most relevant and precise dimension, followed by statistics of applications, and sensors. They also depicted that the best performance is achieved when the three dimensions are combined. Finally, it has been demonstrated that, using voice, the training phase can be reduced from 14 days, used in related work, to five days, without a significant performance detriment.

The paper is structured as follows: Section 2 considers previous related works, divided into three subsections, recent works in continuous authentication, speaker recognition, and a survey about available databases. Section 3 describes the methodology, the architecture of the S3 platform with three configurations, and the dataset collected. Section 4 then shows the results obtained in the different experiments. Finally, Section 5 draws conclusions, and Section 6 sketches possible future work.

## 2. Related Work

This section analyzes the most relevant works regarding users’ authentication, covering different approaches. Although, in order to stay in the scope of the current proposal, the focus will be set on continuous authentication in smartphones, and speaker recognition for user authentication, also in smartphones. Additionally, the review covers also datasets available for these types of problems.

### 2.1. Continuous Authentication

This section reviews several works dealing with continuous authentication. In particular, it focuses on those whose main objective is to develop a system that continuously verifies the user’s identity in a non intrusive way; that is, without the need to repeatedly ask for passwords, tokens, or patterns. In this sense, most of the existing works try to identify the user according to his/her behavior with the mobile device. They do so either by capturing the gestures, the device position, the interaction with it, the use of applications, or a combination of all of them.

For an overview of the different work lines in continuous authentication in mobile devices, the reader can consult [10], where the authors offered an up-to-date and extensive survey, targeted on Behavioral Biometrics and Continuous Authentication technologies for mobile devices. This survey includes an analysis for behavioral biometrics collection methodologies and feature extraction techniques. Finally, the authors also provided another review that showed the vulnerability of machine learning models against well-designed adversarial attack vectors, and they highlighted relevant countermeasures. A list of open challenges in behavioral biometrics can be found in [11]. If the reader is interested in IoT devices, aAn exciting survey can be found in [12], in which the authors present an overview of continuous authentication methods in the IoT environment and include those works based on BlockChain technology.

Continuous authentication uses different kind of data. Data sensors are the most frequently used but many works also consider: applications’ usage statistics, touch screen data, location, and some biometrics such as: facial, voice, and fingerprint, as well. Some of the most recent and interesting works in this sense are described below.

The first work that stands out is SCANet [13]. The authors used sensors data only. In particular, they monitored the device accelerometer and gyroscope to model user behavior. SCANet uses a two-stream Convolutional Neural Network to extract and engineer relevant features, and a One-class Support Vector Machine (SVM) to perform the classification with them. They report that SCANet achieves 90.04% accuracy and 5.14% of EER (Equal Error Rate), using a database they built with 100 volunteers that were asked to carry out three specific tasks: reading, writing and map navigation. In another work [3], a continuous authentication system that uses sensors data and applications usage statistics was proposed. The authors used an Isolation Forest model as a user behavior anomaly detector. This system, in a brief proof-of-concept experiment, is reported to achieve a 92% recall and 77% precision. The Dakota System [14], is another continuous authentication system, for a banking app. This system uses sensors (accelerometer, gyroscope, and magnetometer) and touch screen data. It then uses a One-class SVM to perform classification. The authors created a database of 30 volunteers by collecting their use of the actual bank application, with a minor modification to allow for such collection. The Dakota system achieved 79% of correct user identification, 13% false acceptance and 11.5% EER. Another work that uses touch screen data is [15], where the authors used a Deep Neural Network to make a binary classification.

With regard to the usage of voice, as a dimension in a continuous authentication system, it should be pointed out the aforementioned work done by Feng et al. [4]. In Feng et al. work, the authors designed the IdentityTracker framework, a continuous authentication system that uses touch, voice and motion sensors. The authors indicate that the accuracy is around 95% and 85%, in security and usability, respectively. They also indicate that IdentityTracker may reduce around 85% of unnecessary authentications. They test the framework in a subset of 11 volunteers. Another prior work that uses voice is the one proposed by Shi et al. [5]. In particular, the authors proposed a passive authentication system using voice, location, multitouch, and locomotion. The authors included the voice of voice calls, because in 2011 the use of applications was not yet well developed. They designed the system with voice but, in the data collection and experimentation phase, they did not collect audio, since it is a well-studied field. They did not study how the system behaves. Another, a bit more recent, work that uses voice, is the work by Crawford et al. [8], where the use of voice and keystrokes were the two dimensions selected to build a continuous authentication system. The authors indicated that, in the voice part, they achieved an EER of 25%, and in the key stroke part, EER was around 10%. In the simulation they carried out to test their prototype, they achieved a 67% reduction in the number of authentications required.

As can be seen in the works cited above, it is common to use different kinds of data, with most authors using two or three. Reference [16] stands out for its design, where seven different kinds of data are used: Touch dynamics (touch gestures and keystroking), accelerometer, gyroscope, WiFi, GPS location, and app usage, are all collected during human-mobile interaction to authenticate the users. This system, called MultiLock, use a Support Vector Machine (SVM) with a radial basis function (RBF) kernel to perform the classification and the authors test the MultiLock system in different scenarios in the UMDAA-02 database, reporting an accuracy ranging from 82.2% to 97.1%. Buriro, Conti, and Cripo are authors that work on systems that authenticate the user while answering a cell phone. In particular, their research is focused on authenticating the user by the information contained in the arm movement to place the phone at the ear, and the slide and pick up moves [17,18]. Although they have not developed a proper continuous authentication system, they used the usual data and techniques of continuous authentication in their work, and so it is hereby mentioned. Their last result reports a True Acceptance Rate (TAR) of 85.77% and 99.35%, respectively. Finally, in order to increase security, and to overcome some challenges and weaknesses of the systems seen so far, some works are focused on the design of continuous authentication systems that also use different or additional devices. For example, in [19], the authors proposed the AuthCode System, a continuous multi-device authentication system, for mobile and personal computer. Moreover, in the work [20], the authors used a wearable to authenticate the user by the gait cycles. Table 1 presents a summary of each publication for mobile device reviewed with regard to the Continuous Authentication field.

### 2.2. Datasets Oriented to Continuous Authentication

The latest and most relevant works on continuous authentication, and the published databases, containing relevant kinds of data collected in mobile devices, are summarized below.

Starting with the databases that can be seen in the articles reviewed above, the HMOG Dataset is found [21]. It is a collection that contains real-time touch, sensor and keypress data from three usage scenarios: reading, writing and navigating on a map. A total of 100 volunteers participated in the collection, where each volunteer is expected to perform 24 sessions (eight reading sessions, eight writing sessions, and eight map navigation sessions). In total, each volunteer contributed around two to six hours of behavior traits. In the HMOG Dataset, the attributes recorded are: accelerometer, gyroscope, magnetometer, raw touch event, tap gesture, scale gesture, scroll gesture, fling gesture and key press on virtual keyboard. The HMOG Dataset is available in [22].

Next, the UMDAA-02 Dataset is found [23]. This dataset contains data from the front-facing camera, touchscreen, gyroscope, accelerometer, magnetometer, light sensor, GPS, Bluetooth, WiFi, proximity sensor, temperature sensor and pressure sensor. The data collection application also stored the timestamp of screen lock and unlock events, start and end timestamps of calls, and currently running foreground application. This data set contains data on 48 volunteers on Nexus 5 over two months. The dataset used to be fully available, but not anymore, with only the face and touch part being available now. The website is [24].

Previous databases were collected while users perform specific tasks, or freely uses their device. However, Brain Run Dataset [25] is collected while users play with the mobile. The BrainRun Dataset contains data about touch screen gestures, and sensors information of user when they are playing the BrainRun game. BrainRun is an educational game with a series of different mini-games. The movement data that collected regarding the touch screen are: tap, horizontal swipes and vertical swipes. The sensors data is formed from the accelerometer, gyroscope, magnetometer, and device motion sensor. This dataset also contains information about the different games played by users. The dataset is available and contains 2218 registered users, that is, users who have played at least one game. According to the gathered data, 60% of the users are men, 26% are women, while the rest (14%) have chosen not to reveal their gender. Besides, most users (almost 95%) have only used one specific device to play the game.

All the above datasets contain data from a single type of device, the smartphone. Next, multi-device data sets are going to be reviewed. The SU-AIS BB-MAS, [26] is a dataset collected using desktops, phones, and tablets. This dataset contains data about keystroke, accelerometer, and gyroscope, and also touch screen swipes. The data are collected in different scenarios such as: typing (free and fixed text), or gait (walking, upstairs, and downstairs). The database contains 117 participants during three months. The SU-AIS BB-MAS dataset is publicly available.

Finally, the AuthCode dataset is found [27]. It contains the user’s interactions with personal computers and mobile devices. The dataset was created with five users, who interact for 60 days with their respective devices. The data collected on the personal computer is the activity of the keyboard and the mouse, as well as the statistics of use of the application. For the mobile phone, the collected information includes the sensors and statistics of the use of the applications. A summary of the different datasets can be found in Table 2.

### 2.3. User Authentication Using Voice

Speaker Recognition is a deeply studied field, and a good number of solutions can be found in the literature. In the speaker recognition field different tasks can be researched, such as using text-dependent or independent systems, or using phone audio (8 kHz) or multimedia audio (16 kHz or greater). In the last decade, the emergence of neural networks has motivated research on their use to extract characteristics, or embeddings, that contain information from the speaker [28,29,30].

Currently, the state-of-the-art speaker recognition (SR) audio representation uses an embedding, called x-vector [31], which is extracted from the last or penultimate layer of a Time Delay Neural Network (TDNN), and then, two x-vectors are compared using the Probabilistic Linear Discriminant Analysis (PLDA) [32]. The latest research has focused on finding alternatives, or small improvements to such current state of the art. The authors in [33] used a TDNN-LSTM-Attention neural net, as x-vector extractor with a 6.02% EER, 0.392 minDCF (Detection Cost Function), and 0.395 actDCF, in the SRE19 CTS evaluation. The work presented in [34] shows a system that uses a combination of three different x-vectors extracted from a long short-term memory (LSTM), an extended time-delay neural network (ETDNN) and a factorized TDNN (FTDNN). This three system’s combination achieves the best result of 2.59% EER, 0.153 minDCF, and 0.155 actDCF for the SRE19 CTS evaluation.

The latest works have also looked for techniques to improve the neural network’s performance, developing and testing, a new training method or different loss function. The work in [35] stands out because the authors used an angular margin centroid loss function to train the TDNN, in a try to pull apart more the different user’s classes. The authors report that the AM-Centroid Loss effectively enhances both, the inter-class separability, and the intra-class compactness, of speaker embedding for text-independent SR, when dealing with unseen speakers. Other authors propose changes in the backend process, that is, in the estimation of similarity or distance between two x-vectors. In [36], the author proposes the use a joint Bayesian generative model that replaces the PLDA. The Bayesian generative model reported achieves a 3.142% of EER, 0.3075 for minDCF and 0.4619 for actDCF. It would be interesting to mention recent works where the authors tried to develop a global system for different tasks. The work presented in [37] shows a general system for phone and multimedia audio, using a 2D-CNN trained with wideband and narrowband samples. They report an EER of 3.18% in SITW and 5.44% in NIST SRE16 evaluation, training the system with VoxCeleb 1 and 2 in 16 kHz and SRE in 8 kHz. Finally, it is important to highlight [38], where the author makes an approach through self-supervised techniques. In this way, the author eliminates the amount of tagged data used for training. Restricted Boltzmann Machine (RBM), Autoencoder and siamese network are the techniques used in this work. Table 3 presents a summary of each publication for the speaker recognition detailed previously.

To end this section, and as a main conclusion, it is worth mentioning that voice is not included nor deeply analyzed in the continuous authentication system. There is also no public database that combines both, voice as well as data collected from sensors and statistical applications. Therefore, among the novel contributions this work presents, it can be found: (i) a new framework to continually authenticate the user using voice, sensors, and statistics, (ii) a new database, which contains all kinds of needed data. Besides, this database has not only been collected, but has also been made publicly available at [9].

## 3. Materials and Methods

This section presents the methodology followed in the design and prototyping of the S3 platform. Then, the architecture of the platform, the S3 smartphone application and the S3 framework will be described. Finally, the scenario, as well as the dataset collected and used, are detailed.

### 3.1. Followed Methodology

The long term objective of this work is to design, develop and validate a continuous authentication framework capable of authenticating users when they interact with their device. This particular work is a proof-of-concept of such architecture and the followed methodology is detailed below.

The first aspect was to review state-of-the-art in terms of the work carried out in continuous authentication, and the different attributes and kinds of data that have been used, as well as the databases available. This review has been done (summarized in the previous section) to evaluate the strengths, and weaknesses, of the existing solutions. Once these issues were evaluated, a series of research questions were posed, as outlined in Section 1. As these questions remain unsolved, this work aims to address them or, at least, to alleviate some of the weaknesses identified.

The design of the continuous authentication platform was the next step. First, the focus was set on the selection of the kind of data that would be used in this work: application usage statistics, sensor information, and voice. Next, particular attributes, in each of these kinds of data, were selected. Then, it was designed the app in charge of collecting the data and, finally, the framework itself was designed as well. It is important to mention that some parts of this project rely on a previous work of our research team [3,19]. This allowed for the adaptation and extension of some of its software (for example, the data collection application), and also for helpful discussions that avoided duplication of research efforts, and enhanced the insight about the open research issues of the sensors and statistics based authentication problem.

Once the design was completed, the implementation of the framework was performed. Once done, it was decided to create a database, and to make it public, thus achieving one of the research issues set as goals, which is to provide a research database with these three kind of data to the scientific community. To create such database, a series of volunteers were asked to participate in collecting the data. These volunteers had to install the smartphone application developed, and use their device in a normal way for more than 60 days. It should be noted that this wide time lapse, far longer than the, a priory, expected time lapse needed for competitive training, was made to allow for experimenting with different time windows, another of the goals set for this work. Once the database was collected, and after some pre-process and anonymization of it, the implementation of the framework, and its subsequent deployment on the cloud server, was performed. Finally, experiments to evaluate the performance of the framework were devised and carried out, evaluating several configurations and trying several well known artificial intelligence models. This task was made through a series of experiments to answer the research questions previously mentioned, and obtaining preliminary proof-of-concept evidence of the viability of the framework proposed.

### 3.2. S3 Platform

**Figure 1:**
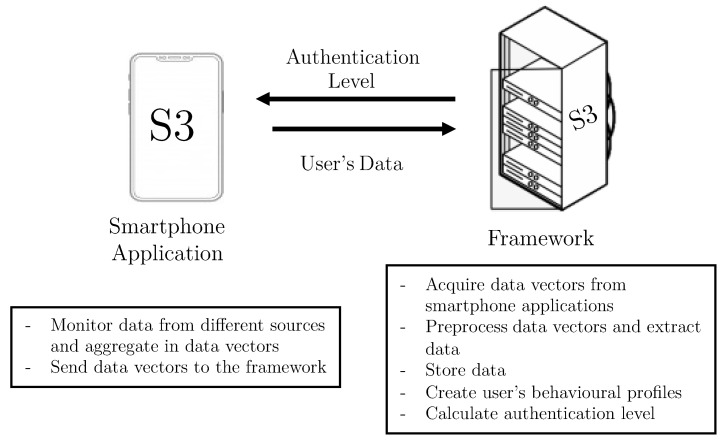
Main parts of the S3 platform and their functionalities.

The S3 platform—Sensors, Statistics, and Speaker Platform—collects data from sensors, applications and voice to create user’s behavioral profiles, that are used to calculate the authentication level of subjects interacting with their mobile devices. This functionality is achieved thanks to a smartphone application, and a framework. Figure 1 depicts the components making up the continuous authentication platform and their main tasks, which are briefly described below.

- Smartphone Application. This component periodically monitors the three dimensions considered in this work: sensors, statistics, and voice. The data monitoring process is carried out by the *Data Acquisition* module, through time windows that are configurable. For the different kinds of data, the attributes are allocated in vectors. These attributes do not contain sensitive user data and are sent directly by the *Connection* module to the framework, deployed in the server side.
- Framework. This is the principal component of the S3 platform. The framework is composed of four modules. Following a bottom-up approach, the *Communication* module has two main functionalities. The first one is to receive vectors with data monitored by the mobile applications. The second is focused on sending the authentication results to the smartphone application, once they have been computed. On top of the Communication module, the *Data Preparation* module pre-processes and sends vectors to be saved in the platform. The *Storage* module creates and maintains the users’ datasets. Finally, the *Intelligent Authentication* module is in charge of: (i) training models containing users’ behavioral profiles, and (ii) evaluating vectors provided by smartphone applications to calculate users’ authentication values in real time.

#### 3.2.1. Smartphone Application

**Figure 2:**
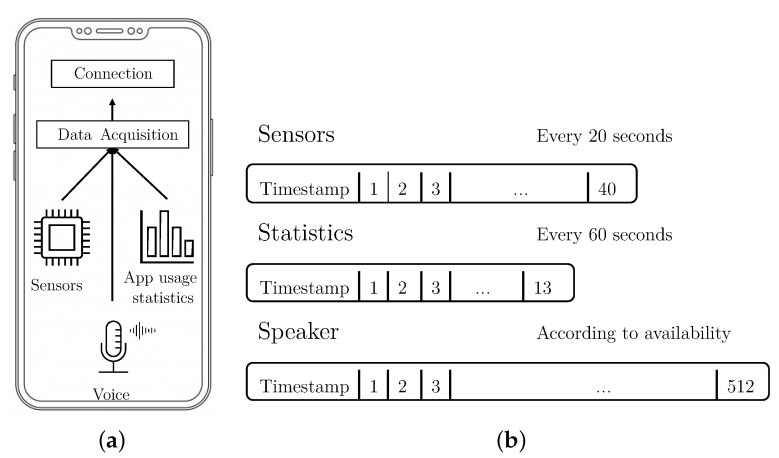
(**a**) Modules making up the Smartphone Application. (**b**) The three data vector extracted by the Data Acquisition module. In the figure, the last number of each vector indicates the number of attributes collected.

The smartphone application is the first component of the S3 Platform, and its objective is to monitor data in real time, to allow the S3 platform to calculate the user’s authentication level. The design and most of the configuration parameters in the smartphone application are based on results from our previous work [3,19]. For the work described in this paper, a new version of the application used in such previous work has been developed. The changes made for this work are: (i) audio capture and process capability to extract the speaker vector, and (ii) modification of the sensor data and statistics capture process to allow it to work as long as the screen is on, instead of only when the device is unlocked. Figure 2a shows a diagram of the application and its two modules.

- Data Acquisition. This module is in charge of acquiring the data from the three selected dimensions: sensors, statistics, and speaker.
- Connection. This module sends periodically the data to the framework.

The Data Acquisition module is in charge of monitoring, at time intervals, or whenever they are produced, the events in each of the selected dimensions: sensors, statistics, and voice. For each dimension, and periodically, this module creates a vector containing behavioral data, and sends the vector to the framework using the Connection module. Vectors of data are sent to the framework with two main objectives. The first one consists of generating the dataset, that will be used to create models of user’s behaviors. The second is to evaluate, in real time, if the user who is interacting with the device is the owner. Figure 2b shows a representation of the three different vectors that the module computes and when they are sampled.

- Sensors vector. This type of vector contains data belonging to smartphone sensors (accelerometer and gyroscope) that has been acquired in a given window of five seconds. The monitored features are:
  – Average of accelerometer and gyroscope values.
  – Maximum and minimum of accelerometer and gyroscope values.
  – Variance of accelerometer and gyroscope values.
  – Peak-to-peak (max-min) of X, Y, Z coordinates.
  – Magnitude for gyroscope and accelerometer [39].
  The process that extracts the sensor vector runs periodically every 20 s, as long as the display is on (as mentioned, this is different from our previous work, where data was only collected when the device was unlocked).
- Statistics vector. These vectors contain data about the different applications used by the user in the last 60 s. Each vector of statistics is calculated every 60 s and contains:
  – Foreground application counters (number of different and total apps) for the last minute and the last day.
  – Most common app ID and the number of usages in the last minute and the last day.
  – ID of the currently active app.
  – ID of the last active app prior to the current one.
  – ID of the application most frequently utilized prior to the current application.
  – Bytes transmitted and received through the network interfaces.
- Speaker vector. This kind of vector is generated when the microphone is active, in a phone call, voice note, or voice command. Each time the microphone is activated, regardless of time, one speaker vector is generated.

Once the application has the collected audio, it is resampled to 16 kHz, if necessary. The application then proceeds to calculate the vector that keeps the information about the speaker. As mentioned in Section 2.3, this vector is called “x-vector”. In order to calculate it, the Kaldi library [40] is used. The process for its calculation is based on Kaldi’s recipe “egs/sitw/v2” and [31], using the models available in [41]. The MFCC (Mel Frequency Cepstral Coefficients) are first calculated, then normalized using CMVN (Cepstral Mean and Variance Normalization) and, as a last pre-processing step, frames of silence are removed by using an energy-based VAD (Voice Activity Detector). Finally, using the neural network available on the kaldi website, the embedding of the penultimate layer of the network is extracted. This embedding, of size 512, is the speaker vector with which the S3 platform works. The attributes of the speaker vector are not interpretable, since the speaker vector attributes are extracted from a trained neural network layer firing, in contrast to the other two vectors, sensors and statistics, whose attributes retain interpretability.

The design and the attributes selected for the sensors and statistics vectors are extracted from our previous work [3,19]. The first attribute of each vector is a timestamp indicating the time when the vector is sampled. Finally, once each one of previous data vectors is generated, the Connection module sends them to the framework.

#### 3.2.2. Framework

The framework is the principal element of the S3 platform. It receives data vectors from the smartphone application, and performs two main tasks. On one hand, it uses artificial intelligence techniques to create users’ behavioral models. On the other hand, it calculates in real time the authentication level of the user interacting with the mobile device. To meet these goals, the framework contains the following four modules:

- Communication. This module receives the data monitored by the smartphone application, and it also sends the authentication results back to third parties.
- Data Preparation. It is where data vectors are pre-processed, and then sent to be stored or processed.
- Storage. This module is in charge of creating and maintaining the datasets for each user.
- Intelligent Authentication. This is the module where the users’ models are trained (in an offline mode), and where the real-time authentication is performed.

**Figure 3:**
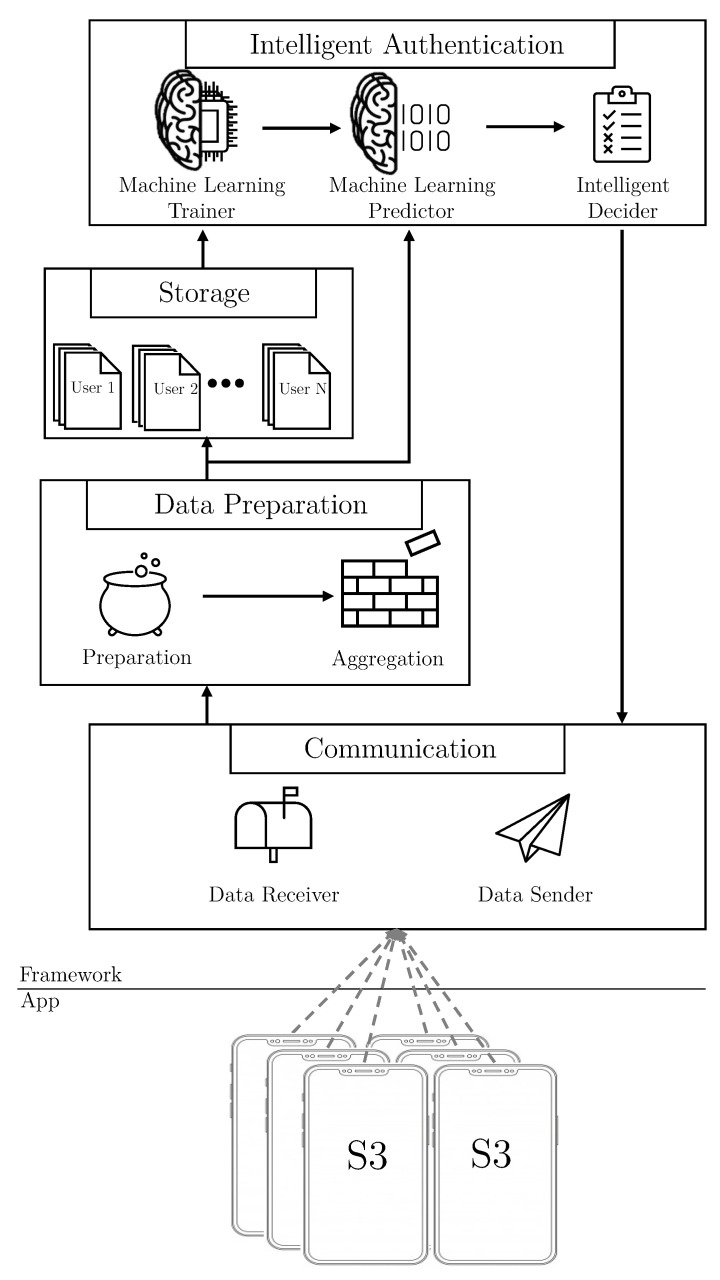
Modules and components making up the S3 framework and its communication flows.

Figure 3 shows, in a schematic way, the modules and components of the S3 framework architecture. The S3 platform is implemented following a distributed approach, where the smartphone application is deployed in the user’s smartphone, and the framework is allocated in a private cloud server.

The communication module comprises two different components to manage the communication with the application and third parties. The components included are: *Data Receiver*, and *Data Sender*, in charge of (i) gathering the data vectors provided by the smartphone applications, and (ii) exposing an API to allow third parties to gather the users’ authentication level.

On top of the previous module, the Data Preparation module applies several pre-processing techniques, such as normalization or curation over the data vectors. In particular, this module implements the following two components to process vectors:

- *Preparation*. This component processes the vectors of data to calculate relevant features, reduce the size of the vectors or do normalization.
- *Aggregation*. In this component, the three kind of vectors: sensors, statistics, and speaker, are combined to form new single “multi-kind-of-data” vectors. This component can be activated or deactivated, in order to form these single vectors containing data from several different dimensions, or to use vectors with only individual dimensions, respectively. The aggregation process considers different time windows established by the administrator, and also deals with the problem of how to aggregate data with different timestamps into a single one.

Once the vectors have been pre-processed and aggregated, the Storage module creates a set of datasets to generate the users’ profiles. The system keeps, for each user, the single kind of data dataset, that is, the sensors, the statistics and the speaker datasets, and also the different aggregations of the combinations of these kind of data, resulting in such a set of datasets for each user. In addition, this module keeps the datasets updated as well, by adding new vectors when it is required.

After the dataset creation, the Intelligent Authentication module has two main tasks: the offline training of the users’ models and the real-time evaluations to calculate the user’s authentication level. To achieve both objectives, it is composed of the following components:

1. *Machine Learning Trainer*. This component generates behavior profiles for each user, using the data from their datasets. It trains, for each user, as many models as different vectors datasets use the framework. It is also capable of training models with different algorithms depending on the particular dataset of a given user. This component runs in a background process, in practice being an offline process, because it is not necessary to be executed in real-time. Therefore, the training process can be scheduled when the server workload is low. When the training finishes, the model is sent to the next component and is ready to be used.
  For each user, the behavior profiles could be generated by the Machine Learning Trainer:
  1. Using vectors of a single kind data:
    (a) Sensors
    (b) Statistics
    (c) Speaker
  2. Combining vectors from different kinds of data:
    (a) Sensors and Statistics
    (b) Sensors and Speaker
    (c) Statistics and Speaker
    (d) Sensors, Statistics, and speaker

- *Machine Learning Predictor*. When the Data Preparation Module sends a vector, this component evaluate it with the respective user model to calculate the authentication level. It is important to note that the calculation of the authentication level is done in a real-time process.
- *Intelligent Decider*. This component computes the final authentication level of users, implementing the following three alternative configurations:
  – The *simple* configuration, where the score received by the Predictor component is directly sent to the Data Sender without modification.
  – The *combination* configuration, where a new score is calculated combining previous scores from other dimensions.
  – The *smooth* configuration, where the score is smoothed with previous scoring to avoid erratic behavior.

#### 3.2.3. Framework Deployment

Once all the modules and the components of the framework have been explained, the three forms of configuration that the framework presents will be detailed below.

As previously mentioned, three different kinds of data vectors reach the framework at different times and frequency. In particular, Sensors vectors are collected every 20 s, Statistics vectors every 60 s, and Speaker vectors whenever voice information is available. Each vector contains a timestamp, allowing their aggregation according to time windows. This aggregation can be done at two levels:

(i) At the vector level. The available vectors (one for each kind of data) are aggregated into a single vector. Then, a user’s model that has, as input, one of such aggregated-vectors is evaluated and a score produced.
(ii) At score level. One user model per kind of data is evaluated separately, and then, the score of each model is combined into a single final score value. This type of aggregation is termed “score combination”, to differentiate both types of aggregations.

The framework design thus allows for three configurations: without aggregation, with score combination, and with vector aggregation.

**Figure 4:**
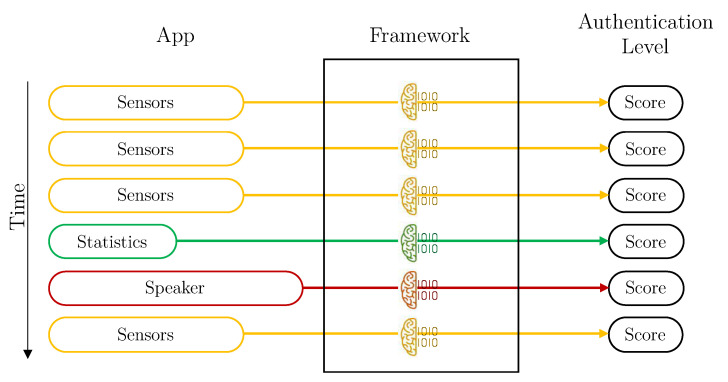
Vector process flow for the deployment without aggregation, each color represents a different kind of data (yellow: Sensors, green: Statistics, red: Speaker).

*Without aggregation* is the simplest design. The framework calculates the user’s authentication level for each vector of data received. In this case, the Aggregation component of the Data Preparation module is deactivated, and the Intelligent Decider is in the simplest mode. Therefore, each user has three individual machine learning models trained: sensors, statistics and speaker. Each time a vector is available, the score is predicted with its corresponding model, and it is directly used. Figure 4 depicts how vectors are managed by the Framework in this configuration.

In *Score combination* configuration the Intelligent Decider is in combination mode, and the vector Aggregation component is not used. Now, the score is calculated using a combination of the previous scores. The calculation of the combined score is performed as follows There are three variables that store the latest scores for each vector type, $score_{sen}$, $score_{sta}$ and $score_{spe}$, and another three variables that store the timestamp of each vector for which these latest scores have been calculated ($time_{sen}$, $time_{sta}$, and $time_{spe}$). When the Intelligent Decider checks if the timestamps are in a time window, the following cases can occur:

1. The three vector scores (one per dimension) are within the current time window. The authentication level is combined as:
  (1) $$Level_{t}=\alpha *score_{sen}+\beta *score_{sta}+\gamma *score_{spe}$$
  where α+β+γ=1.
2. Two of the timestamp are in the time window. The level authentication is defined as:
  (2) $$Level_{t}=\alpha_{xy}*score_{x}+\left(1-\alpha_{xy}\right)*score_{y}$$
  where $\alpha_{xy}\in \left[0,1\right]$ and x,y∈{sen,sta,spe}.
3. Only a timestamp is within the window:
  (3) $$Level_{t}=score_{x}$$
  where x∈{sen,sta,spe}.

**Figure 5:**
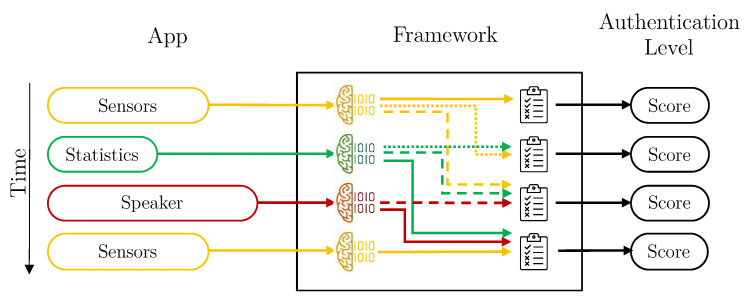
Vector process flow for the deployment with score aggregation, each color represents a different kind of data (yellow: Sensors, green: Statistics, red: Speaker).

Figure 5 depicts a graphical representation of how the different vectors arrive at the framework from the application, and how they are processed. In this case (a combination of scores), the vectors are evaluated as they arrive, each with its corresponding model. Once the score has been obtained, it is combined with other previous scores, in the Intelligent Decider, to obtain the final authentication value.

*Vector aggregation* creates new vectors, grouping the values of all kind of data attributes. That is, it creates “multi-kind-of-data” vectors, to be used to train a single model using these aggregated vectors. In this case, the Aggregation Component from the Data Preparation Module is active, and the Intelligent Decider is set to simple mode. It is important to note that, the vectors aggregation, can only take place if the timestamps of these vectors are in the same time window. If that is not the case, the aggregation component uses only the recently received vector.

**Figure 6:**
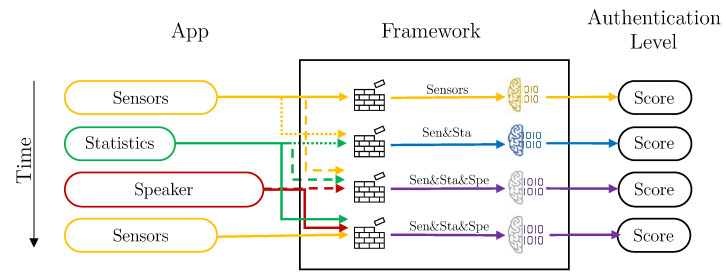
Framework process flow for the deployment with vector aggregation, each color represents a different kind of data (yellow: Sensors, green: Statistics, red: Speaker, blue: Sensors and Statistics, and purple: sensors, statistics, and speaker).

Depicting with the same example vectors as in Figure 5, Figure 6 shows how the system would work using vector aggregation. In this case, once the vectors reach the framework, the aggregation component checks if there are other vectors for other dimensions in the same time window and, if so, they are grouped to form a vector with all attributes together. Once they are grouped, they are evaluated with the specific model of each kind combination vector.

### 3.3. Scenario and Dataset

To demonstrate the feasibility of the S3 platform and to improve the state-of-the-art in terms of dataset, for continuous authentication combining voice, sensors, and statistical data, a dataset has been created, and made publicly available in [9]. The dataset contains the behavior (sensors, statistics of applications, and voice) of 21 volunteers interacting with their smartphones for more than 60 days. The type of users is diverse. Males and females, in the age range from 18 until 70, have been considered in the dataset generation. The wide range of age is a key aspect due to the impact of age in terms of smartphone usage.

To generate the dataset, the volunteers installed a prototype of the smartphone application on their Android mobile phones. At this point, it is important to mention that the implemented prototype is not available for other platforms, such as iOS. During the monitoring phase, the users could use their smartphone freely, without restriction, as they would use it normally. The application automatically monitored the sensors values, statistical information from applications and audio records. Regarding the audio collection, to avoid obtaining sensitive and personal data, a series of texts were established in advance. Each day, the participants received, or made, one call, where a particular text (from the list of available ones) was selected, read and recorded. Each participant received, or made, only one call per day, from Monday to Friday, for six weeks, holidays excluded. In addition, every day, participants received a text message with some sentences that should be read, and recorded, as voice messages. Table 4 shows a summary of the details of the collected database.

**Figure 7:**
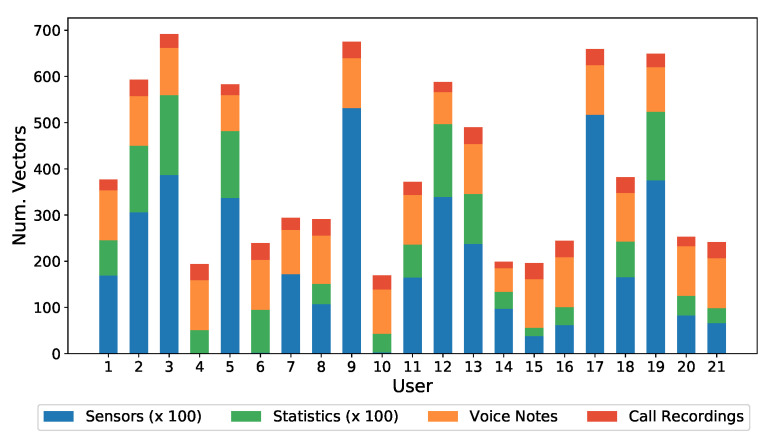
Dataset distribution by user. For voice notes and call recording data, the values are in real scale, while for sensors and statistics data, the scale is 1:100.

Figure 7 shows individual (per subject) distribution of the different kinds of data contained in the dataset, with Voice Notes and Call Recordings (both being Voice kind of data) desegregated. From the sensor and statistical data it can be seen how there are three clearly differentiated groups of users, depending on whether they made heavy or light use of the device. Users 2, 3, 5, 9, 12, 13, 17, and 19 made heavy use of the device, while users 14, 15, 16, 20, and 21 made light use of it, with less than 50 h in two months. With regard to audio samples, all users have a similar number of samples.

Note that some users do not have some kinds of data. For example, users 4, 6, and 10 do not have sensor data, because their mobile phones models did not have such sensors. In any case, it was decided to maintain this type of user in the database, because this is a feasible situation in a real deployment, as it happened to be in this data acquisition.

## 4. Results

This section presents a set of experiments that explores the performance of the S3 platform, evaluates the relevance of each dimension and assesses the viability of the S3 platform as a continuous authentication system, being the collected evidence a first proof-of-concept. The section is organized as follows. First, the common details of all experiments, and the metrics used in them, are provided. After that, the first experiment is described, which consists in exploring, among several anomaly detection algorithms, which one shows the best authentication result using a single dimension (sensors, statistics, or speaker). Next, the second experiment also explores the best results among several unsupervised algorithms, but this time using the aggregated vectors, combining data from different sources at the same time. The third experiment studies how the number of days of training data affects the S3 platform performance. Finally, in the fourth and last experiment, the S3 platform results are evaluated from global perspectives.

### 4.1. User Profiling Approach

As previously mentioned, the approach followed to characterize the users’ behavior has been to consider it as an anomaly detection problem. For this consideration, scalability of the solution has been taken into account, and the worst case scenario regarding training data available has been considered, that is, a scenario where only data of the user’s behavior is available for training. Therefore, in these experiments, an unsupervised learning approach to anomaly detection will be explored. Hence, users’ models and behavior would be obtained using various unsupervised Machine Learning techniques.

In addition to the different anomaly detectors, the cosine score and the PLDA have been used for the speaker vector. The cosine score is an unsupervised technique, while the PLDA is supervised, as it needs to be trained with labeled data. However, in the experiments presented in this work, the PLDA available in [41] is used, which has been previously trained with an independent database. In other words, the data of the users of the S3 platform have not been used to train the PLDA used here. Therefore, in that sense, it can be considered that the extraction of the user profiles used in S3 still follows an unsupervised approach. The general methodology that was used to do the experiments, and the evaluation metrics used are explained below.

#### 4.1.1. General Methodology

The objective of the S3 platform is to perform user authentication by evaluating user behaviors. For this, user behavior profiles are generated in order to compare them with future new data samples, and so detect if such samples show a suspicious behavior that does not correspond to the profiled user. A signature-based approach is not an option, as the objective is that the profiling would be accomplished using automatic artificial intelligence techniques. It was also considered preferable to work in a worst case scenario, where only data from a single user could be used for profiling. Privacy issues may, for example, force the learning to be made in the own device, in order to avoid any sensible data going to the cloud or servers. This is not expected to be the case when S3 reaches operation stage, as encryption or anonymizing data techniques can be used with really sensible data, and still be safely stored and used on the cloud servers. Nonetheless, at this stage of proof-of-concept, it is worth checking the system’s capabilities when all conditions are adverse. Therefore, no labeled data will be used in training, and an Anomaly Detection approach, with models obtained by unsupervised Machine Learning algorithms, was the base of the experimentation at this stage of the research.

Following this approach, the dataset collected, see Section 3.3, has been split into train/test for each user. Data from the first 14 days were used to train the user models, each user with its own first 14 days of data only.

On the other hand, how to evaluate the performance of the models obtained was considered. As no data of users trying to impersonate individuals were collected, or available, using the closest data available to take that role was considered, that is, using the data of all other users as contrast, and hence evaluate whether S3 can differentiate the rest of the days of data from the same user (tagged as positive) from the rest of data of all the other users (tagged as negative). Therefore, for each user, a test set is created where, from the 15th day until the last day of the user, collected data are tagged with a positive label and, for negative labels, all data from the rest of the users. This way, metrics of classification problems can be obtained to evaluate the performance. This configuration was decided based on the previous work of part of the research team in [3,19]. Although individual users’ models have not been compared in this work, it should be noted that each user has a different test set.

An exception to this 14 day training set is found in experiment 3, in Section 4.4, where the objective is to evaluate the S3 performance varying the number of days available for training. In that experiment, the number of days of a single user used for training and to generate the users’ profiles vary. Namely: 1, 2, 5, 7, 14, and 21 days, and the corresponding data from 2, 3, 6, 8, 15, and 22 days, until the last day of collected data, were used to build the testing sets. Note that the performance using less, and more than the 14 days, has been explored.

For each user, the vectors are normalized using the Standard Scaler, canceling the mean and scaling to unit variance. The normalization parameters are obtained from the train split, and applied to all vectors in both, the training, and the test set. In a previous phase, different types of normalization were tested, such as the Standard, MinMax and others, but no significant statistical difference among them was found.

Several unsupervised Machine Learning Algorithms have been evaluated during the preliminary phase of experimentation, and the systems with the best performance were: the Isolation Forest (IF), the k-Nearest Neighbours Algorithms (KNN), and the Angle-based Outlier Detector (ABOD). These were the ones selected for use in the experiments.

Once the dataset is split in train/test for each user, the above mentioned machine learning algorithms are used to extract the user behavior with the training set. Once trained, the test set is evaluated against the model. These algorithms work as outlier detectors, and score new data regarding the confidence of such data to belong to the training data distribution. This evaluation obtains a score that is normalized to the zero-hundred range. The highest scores are associated with the positive class, that is, to be the same user, in the test sample, as in the trained model.

As mentioned, when the models used are evaluated a score is obtained, which is interpreted as a probability or similarity value. Higher scores indicate more similarity between the users’ profiles and the test sample. In the training phase, the normalization needed to transform the score to the zero-hundred range is calculated. To decide if a new sample is classified as positive (the user is authenticated) it is needed to fix a threshold and check if the score is greater or lower than such a threshold. In most cases, the selected threshold aims to obtain the highest precision or accuracy but, in an authentication problem, usually a maximum false alarm probability that the system must comply with is set. As will be mentioned later, when measuring the performance, the metrics are selected to be threshold-independent.

When all user models are trained and evaluated with their correspondent test split, the results of each user model are obtained. All user results are combined to calculate the metrics explained below. In this way, the metrics, in fact, represent a measure of the global performance of the whole system. Of course, all these metrics can be calculated individually for each of the platform’s users but, here, the whole platform performance is the key interest. Therefore, and unless otherwise specified, the metrics and results shown below are always carried out globally for all users.

#### 4.1.2. Used Metrics

As mentioned above, in order to evaluate the performance, a confusion matrix is calculated using the test data with its positive and negative tagged data, counting the number of cases correctly assigned (true positives and true negatives) and the number of cases where the system gives a wrong authentication (false positives and false negatives). As shown in Table 5.

With the above basic figures, the selected metrics to evaluate the performance of the S3 platform, are the following:

- AUC: the Area Under the Curve measures the two-dimensional area underneath the entire Receiver Operating Characteristic (ROC) curve. Such ROC curve can be used to estimate the performance, as it depicts all combinations of sensitivity and specificity (in general, increases in one means decreases in the other), since the ROC draws the ratios of true positives (sensitivity) versus ratio of false positives (one minus specificity).
  $$Sensitivity=TPR\left(\mathrm{True}\mathrm{Positive}\mathrm{Rate}\right)=\frac{TP}{TP+FN}$$
  $$Specificity=1-FPR\left(\mathrm{False}\mathrm{Positive}\mathrm{Rate}\right)=1-\frac{FP}{FP+TN}.$$
- EER: the Equal Error Rate is the value where the false acceptance rate (FAR), and the false rejection rate (FRR), are equal.
  $$FAR=FPR\left(\mathrm{False}\mathrm{Positive}\mathrm{Rate}\right)=\frac{FP}{FP+TN}$$
  $$FRR=FNR\left(\mathrm{False}\mathrm{Negative}\mathrm{Rate}\right)=\frac{FN}{TP+FN}.$$
- F1-max: is the maximum of the F1-score. The F1-score is the harmonic mean of precision and recall (TPR), and is calculated as follow:
  $$Precision=\frac{TP}{TP+FP}$$
  $$Recall=TPR\left(\mathrm{True}\mathrm{Positive}\mathrm{Rate}\right)=\frac{TP}{TP+FN}$$
  $$F1=2*\frac{Precision*Recall}{Precision+Recall}.$$

### 4.2. Experiment 1: Continuous Authentication with Isolated Sensors, Application Statistics, and Voice

The objective of this experiment is to assess the continuous authentication system using only one kind of data vectors (the “without aggregation” configuration from the S3 platform), and to explore the behavior of several anomaly detection Machine Learning Algorithms. In this sense, it will be determined the capacity of each dimension to authenticate users, paving the way to answer research question number one outlined in Section 1.

#### 4.2.1. Data

For this, and the rest of experiments, the database, collected as detailed in the previous section, is used.

In particular, data used in this first experiment are:

- Sensors vectors, the 40 attributed indicated previously, plus the weekday (in categorical form, from 1 for Monday to 7 for Sunday) and the number of seconds in the day. Therefore the sensors vectors are vectors with 42 attributes.
- Statistics vectors, the 13 attributes of apps usage statistics, plus the same two attributes added to sensors vectors. The name of the apps used in the vectors was coded as categories, and is finally coded as a whole number. The statistics vectors are vectors with 15 attributes.
- Speaker vectors, the 512 values of the x-vectors. The speaker vectors are vectors with 512 attributes.

Each of these vectors has a timestamp of when it was generated but this variable is not used when the model is trained or evaluated.

#### 4.2.2. Models

To determine which algorithm to use, for the continuous authentication using only one vector type, three algorithms will be evaluated.

For the sensors and statistics vectors the selected algorithms to be evaluated are: Isolation Forest (IF), k-Nearest Neighbors Detector (KNN), and Angle-based Outlier Detector (ABOD). These have been selected by a previous exploratory screening. The PyDot Python library [42] will be used as the implementation of these algorithms, to facilitate independent replication. A brief sweep on their main algorithm parameters (for example, size) was explored. It is well known that, rather frequently, the machine learning algorithm’s performance is very sensitive to the algorithm parameters used but, at this stage, a good enough result is what is sought. Future work would aim to further optimize these parameters in an effort to obtain the best models possible. However, that is beyond the scope of this work, which mainly attempts to collect evidence and clarify whether it is promising and worthwhile to add voice to continuous authentication systems.

For the speaker vectors, the current state-of-the-art algorithm and parameters will be used—the PLDA, the Cosine Score Metric and the Isolation Forest. When IF is tested, the same normalization as described for sensors and statistics is used. When the cosine score function is used, the normalization was applied, and for the PLDA the steps from the Kaldi’s script are followed using the PLDA models available in [41].

#### 4.2.3. Evaluation

**Figure 8:**
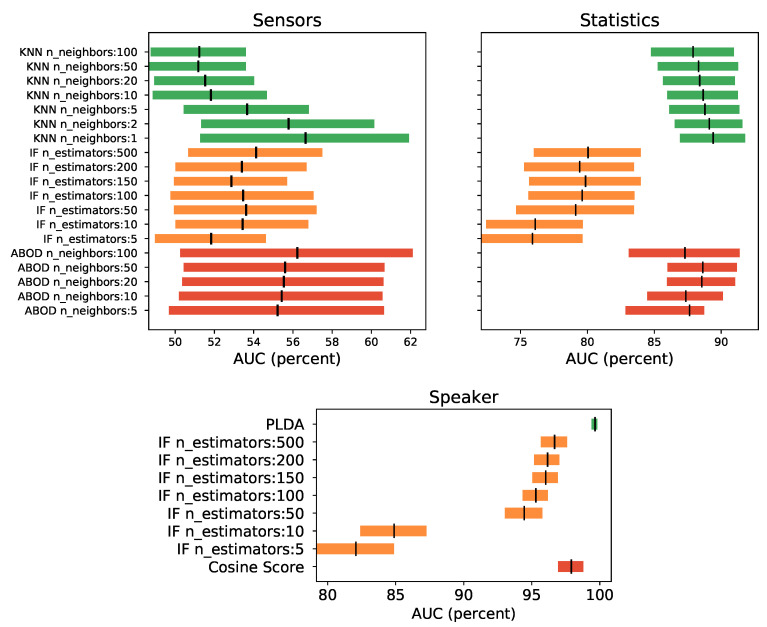
Confidence intervals for the results of selecting the best individuals models for sensors (left), statistics (right), and speaker (down) vectors. The algorithms selected are KNN (green), IF (orange) and ABOD (red) for sensors and statistics and IF (orange), cosine score (red) and PLDA (green) for speaker vectors. The black line is the mean of the interval confidence.

To evaluate the different algorithms, the metrics of Section 4.1.2 are used. To show significant statistical difference between experiments, the confidence intervals results for the best model among all users (not the global system measure) are drawn in Figure 8. These results show that, among the algorithms tested, the one that provided better performance was the KNN for both kinds of data vectors—sensors and statistics—although for the sensors case it shows no significant difference among the three algorithms. Likewise, for statistics vectors, KNN and ABOD show no significant differences as well. In both cases, the slightly better behaved model (highest low interval value) was the KNN with only one neighbor. It should also be noted that models trained with the statistics dimension provided better results than the sensors model, by a large amount, with near to 90% versus 56% of AUC respectively. Actually, sensors models have quite poor AUC values and hence quite poor performance. For the speaker vectors, it can be seen in the graph below Figure 8 how the PLDA is the algorithm with the highest AUC value. In addition, it can be seen how its confidence intervals do not intersect with those of the rest of the algorithms, clearly indicating that it is the best algorithm of those evaluated.

Nevertheless, a better estimation of the performance of the global system would be obtained with a global metric that considered all the user models of the system as one, sharing the same threshold. These global metrics for all user models using KNN with one neighbor are provided in Table 6.

The results for the single dimension models with the speaker vectors are in Table 7, where it can be seen that the tested algorithm with a better performance is the PLDA. PLDA technique improves the AUC metric by almost two whole points to the second best voice algorithm, by more than 5 points using the EER metric, also to the second best, and by more than 20 with the F1-max metric. In the same table, the last two rows show the results obtained by the sensor and statistical systems trained with all its data but evaluated, obviously, with its own vectors, but only with data at the times where voice is present (about the same timestamp than the test voice vectors used to evaluate those metrics in order to make a fairer comparison). This way, it is easy to see that, single models that use only sensors and statistics kind of data perform worse than the use of single model trained with the speaker vector. It is interesting to point out that the results obtained in these situations (when voice is present), with sensors and statistics models, are worse than the overall results for such models in Table 6. In these situations, sensors detection performance decreases dramatically and the statistics are reduced in three points in AUC and EER. It is hypothesized that such a decrease in sensor detection may be related to the fact that the movements, when the phone is used to talk, may be substantially different to those during other activities, and/or that these situations are far less frequent. When this is so, due to data unbalancing, the outlier detection unsupervised machine learning algorithms may fail to characterize these situations as non-outlier. Nevertheless, the test of such a hypothesis is out of the scope of this work.

According to the results obtained in this experiment, the voice dimension provides a lot of information for the authentication of the users in the moments in which it is present. Furthermore, it has been seen how the sensor and statistical systems significantly decrease their normal performance precisely in these situations, worsening their results quite dramatically in the case of sensors, up to a point of rendering them unusable, and thus providing a solid argument to include voice in this kind of authentication systems.

In order to compare these results with the next experiment, the KNN with one neighbor algorithm will be the chosen machine learning algorithm to be used for the sensors and statistics vectors, and the PLDA for the speaker vectors.

When these results are compared with those seen in Section 2, it can be seen how the SCANet system, using only data from the sensors, obtained much better results, a 5.14% EER compared to the 39% shown here. Such a comparison is not really fair, however, since SCANet uses a database with a limited range of activities, in particular: reading, writing and navigating on a map. This short range of activities made an unrealistic scenario, and simplifies the authentication very much. The database collected and used in this work is more difficult, because it includes a real world scenario.

The result of the models with only statistics vectors is better than only with sensors vectors, and it cannot be compared directly with other systems found in the literature because none of such systems uses statistic information alone. Again, the voice system can not be directly compared, as well, since this kind of models can not be fairly compared directly when using different databases. Nevertheless, it can be seen that the results of systems found in the literature are in the range of the work presented here.

### 4.3. Experiment 2: Continuous Authentication Combining Sensors Application Statistics, and Voice

The objective of this experiment is to evaluate how the S3 framework performs when combining different kinds of data vectors, and to answer research question number two outlined in Section 1. In order to combine the data, the two options detailed in Section 3.2.3, combining scores, and aggregating into a single vector, are explored.

Two aggregations will be tested: sensors and statistics on one side, and sensors, statistics and speaker on the other. Other combinations of vectors have not been used because they do not occur in reality. Whenever sensors information is available, statistics are also available, and vice versa.

#### 4.3.1. Data

The data used in this experiment are the data used in the first experiment with some minor changes:

- Sen&Sta vectors, the 40 attributes from sensors vector and the 13 attributes from the statistics vectors. Again, the weekday (in categorical form, from 1 for Monday to 7 for Sunday) and the number of seconds in the day are added to the vector. Thus, these vectors have 55 attributes.
- Sen&Sta&Spe vectors, for this vectors it is used the 40 feats from sensors, 13 from the statistics, and 512 feats from the speaker, also it is added the weekday and the number of seconds. The vectors have 557 feats.

To create the aggregated vectors for this experiment, a window of 60 s is set in the configuration of the framework. This means that each time a new vector is received of any kind, the last vector of each other dimension is added to it, only if it was received less than 60 s ago.

#### 4.3.2. Evaluating Sensor and Statistics

The results about the two different aggregation methods can be found in Table 8. In this table, the two first rows show the results obtained by the individuals models (sensors and statistics) trained with all its data, but evaluated here with a subset of its test data, including only instances where both sensors and statistics vectors are present in the same time window, that is, the same test instances that, combined, form the Sen&Sta model test set, and hence the different results for these models in this table to those shown in Table 7.

The third row shows the results for the “score combination” method, a linear combination of individual scores using Equation (2), after selecting the best results trying different combinations of factors. The best results found were with $\alpha_{best}=0$. This implied that the score combination, in fact, it is the same as only use the statistics score.

The last row of the table shows the “vector aggregation”. In this case, a KNN is used with a neighbors parameter equal to 1 to evaluate the behavior of using the aggregated “Sen&Sta” vectors. This was determined because in the previous experiment the best individual models for sensors and statistics was that algorithm with such parameters and so it would be a fairer comparison. The vector aggregation gets results close to the use of statistics individually for the AUC metrics, but in the case of the F1-max, this aggregated vector model achieves better performance than the rest.

#### 4.3.3. Evaluating Sensor, Statistics and Speaker

Next, the aggregation of all the available information: sensors, statistics and speaker, is going to be evaluated. For the “vector aggregation” method, again to make a fairer comparison, the selected algorithm is the KNN with neighbors parameter equal to one. For the “score combination” it has been carried out a linear combination of the three scores using Equation (1), again selecting the best results of several combinations tried. The best result was obtained for the following combination of parameters:

(4) $$\alpha_{sen}=0.0\;\;\alpha_{sta}=0.8\;\;\alpha_{spe}=0.2$$

Table 9 show the results for these two approaches. The first row of the table also contains the best results for the voice single model using the PLDA technique, to ease comparison. As has been done previously, the voice model is evaluated using a test set that includes only data where the three vectors are available in the same time window. In this experiment, the best result is the *Score Combination*, that performs only marginally better in all metrics versus the voice system. The system trained with the Sen&Sta&Spe vector shows rather worse results compared to the other two.

The excellent result of the voice with the use of the PLDA algorithm completely overshadows this experiment. Before the evaluation, it was hypothesized that, given this kind of precision in the Voice only models, the combination of scores with individual sensors and/or statistics, trained with non optimized unsupervised outlier detection learning algorithms, may not provide any useful information, and would end up as worse or, at most, equal systems (with a combination $\alpha_{sen}=0,\alpha_{sta}=0,\alpha_{spe}=1$, for example). However, as can be seen, the results of combining the scores improve the result obtained, with the statistics score providing 80% of the score value.

From experiments 1 and 2, it easy to see that the voice, by itself, achieves excellent results. However, it has to be reminded that it alone cannot constitute a continuous authentication system. This is so because the voice is only present on rare occasions, when there is a phone call, or the user sends an audio note. Therefore, it is necessary to have other dimensions, such as sensors and statistics, with a much higher frequency in time and these dimensions can never be overlooked.

As a final note, aggregating all kinds of data in a single vector has shown bad results compared with a combination of scores. To be fair it should be reminded that the algorithms used are outlier detector unsupervised machine learning algorithms and adding extra attributes to these problems would lead to more complex characterizations in hyperspaces with no counter-examples. When, in the future, supervised learning techniques would be tried it is expected that such under performance may be reduced or even reversed.

### 4.4. Experiment 3: Performance Evolution with Regard of Time Window Data Used for Training

One of the biggest challenges in continuous authentication is getting very accurate systems without requiring many days of training data. In the two first experiments, the number of days to train was fixed to 14 days. This number was selected following previous works and seeming a sensible lapse that would provide enough data information to generate acceptable user profiles. Nevertheless, 14 days may be, in fact, a lot of days without continuous authentication. Therefore, and because training time window is also a hiper-parameter to be optimized, it was decided to evaluate how the individuals models improve their performance regarding the number of training days, to collect evidence as to when the algorithms starts to reach an acceptable performance, and when they plateau in its improving with new data. This experiment, therefore, answers research question number three.

#### 4.4.1. Data

In previous experiments, it was determined that it is better to use individual models for each of the dimensions, and then combine their scores, than using aggregated single large vectors. Therefore, for this experiment, no aggregated model was tested, only single kind of data models, and therefore the same data as in experiment 1 will be used.

#### 4.4.2. Models

Again, following results in the first experiment, it will be trained a KNN model with a neighbor parameter equal to one for the sensor and statistical vectors, while for the speaker vector, the PLDA algorithm will be used. These models are the ones that have obtained the best results so far.

As it was said in Section 4.1.1, for each user, the first days of collected data will be used to train the models and the rest for tests. For each user, the amount of days of data evaluated are: 1, 2, 5, 7, and 21 (besides 14, previously evaluated in experiment 1).

#### 4.4.3. Evaluation

**Figure 9:**
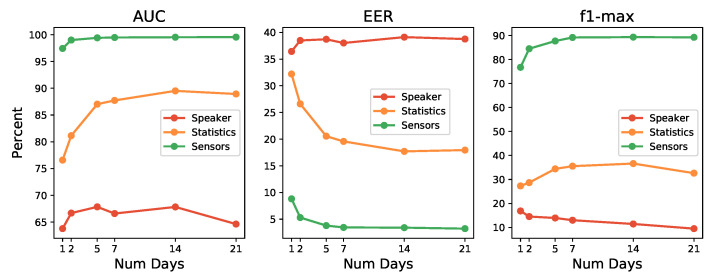
Metrics’ Evolution when increase the number of day for the training step.

Figure 9 shows the evolution of the performance metrics as the days of data collected used for training increase. It is interesting to observe the fluctuating behavior of the sensors models (red line), where the best for the AUC metric is reached with five days, then down and then up again (but inside the error boundaries), while for the EER (remember that for EER, smaller values means better models), best models need only a single day. For the F1-max metric, the performance decreases as the number of days increases.

Results for sensors seem to point that the algorithms used do not seem to be able to achieve a good characterization of users. Algorithms tend to increase performance as more data is available (up to certain point, and always with an eye set to avoid overtraining). When that does not happens, and metrics keep giving low performance, it may be signs that the algorithms used may not be suitable for that particular data set. If that is the case, other machine learning techniques with other in-built biases, should be explored. Nevertheless, on further inspection, the decrease produced from day five to seven for the sensors case, which was rather discouraging, revealed that day six and seven correspond to Saturday and Sunday. This suggests that users interact differently with their mobile devices on weekends or, at least, the postures and positions of use vary with respect to the working days of the week. Taking all of this into account, in future work, it may be interesting to explore data segregation or train particular models for these two types of days of the week, or to particularize the models by the activities carried out by the user before completely discarding the tested algorithms. If that is not enough, it would be wise to take these issues into consideration if other learning algorithms are applied in search of one machine learning technique that can characterize users with sensor data with better performance than the one achieved here.

On the other hand, statistics and speakers vectors graphics show a more learning-effective behavior, improving their results as the number of data days for training increases. For statistics vectors, the best result is achieved with 14 days, which is better than for 7 and 21 days for the different metrics. For the speaker vectors, it improves continuously with the number of days data, but the improvement produced is soon rather small.

These results verified that the use of 14 days to train the user models was an adequate and sensible choice. Besides, it has been possible to verify that, by using only 5 days, the performance reaches a value very close to that of 14 days. This fact suggests that the S3 platform may begin to be operative and return an acceptable authentication level from day 5 of collecting data on the user. The system will improve, of course, if it is allowed to keep training until 2 weeks of use.

### 4.5. Final Proof-of-Concept Performance

Finally, after analyzing the results obtained in the previous experiments, the final deployment configuration for the S3 framework decided was the “score combination”. In this configuration, each user has only three profiles, one for each kind of data, and the authentication level is calculated by combining the scores from the individual models. Section 3.2.3 provides more detail about this configuration.

Once the configuration for the framework has been elucidated, the final proof-of-concept evaluation of the S3 platform functioning can be determined. In security, the two most important factors to be taken into account to assess a system are: False Positive Ratio and False Negative Ratio. The FPR, in the sense that for a system to be considered usable a maximum FPR must be ensured, being a measure of the frequency of checks of the authentication system, would tag as the real user another user. Such a maximum should be established and the algorithm threshold used to decide outliers should ensure such a maximum. To evaluate the behavior of the S3 platform, two work points are set. The two FPRs where the S3 platform will be evaluated are 30% and 10%. Once the minimum FPR is set, the thresholds for the algorithm output values to be considered as a positive or negative results can be calculated. With these thresholds the False Negative Ratio can then finally be calculated, which would measure how often the authentication method would raise a false alarm (and possibly request an action) while the real user is using the mobile. The thresholds and the FNR value reached for these two works point are listed in Table 10.

**Figure 10:**
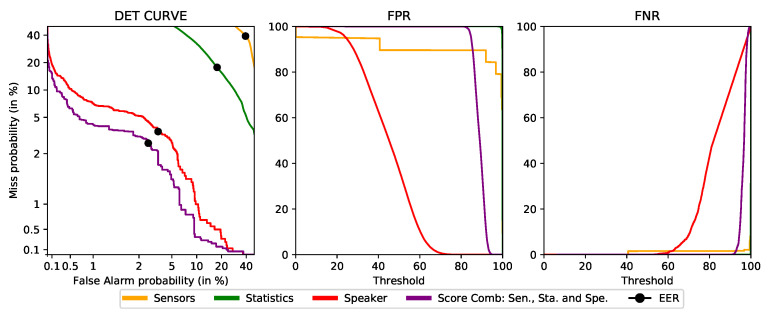
DET Curve, FPR and FNR curves before the score calibration.

As can be seen in the Table, except for the cases where there is voice, all the thresholds are excessively high; the lowest is 99.98. Taking into account that the score moves in the range 0–100, it implies that the models have too little resolution to adjust the working threshold. Observing the FNRs, one can see completely different behaviors from the sensor system that would prevent access to the legitimate user on too many occasions, to the combination of the three types of data, which would work ideally. In more detail, it can be seen in Figure 10.

Figure 10 depicts how the system behaves when the minimum ensured FPR for the S3 platform varies, using a Detection Error Tradeoff curve (DET). DET curves are widely used in the field of authentication, and they follow a similar rationale as ROC curves, but instead of True Positive Rate (or sensitivity), it is plotted the False Negative Rate, and it is plotted against the False Positive Rate (or one minus specificity), the same as in ROC. Figure shows as well the FPR and FNR metrics with respect to the threshold. In the graphs from the FPR and FNR plots, it can be seen that there is very little resolution with respect to the thresholds detailed in Table 10. A small change in the threshold can cause the metrics to vary enormously. Even more, the same threshold has very different results for the different vectors that the system uses. To avoid this, a calibration function that translates the original scores to others with higher resolution was decided to be applied for each vector.

The calibration function applied to the score that has been selected is the interpolation of the following points:

$$f\left(threshold_{@\mathrm{FPR}=X\%}\right)=100-X;\;\mathrm{where}\;X=\left[0,1,2,3,\cdots ,100\right].$$

**Figure 11:**
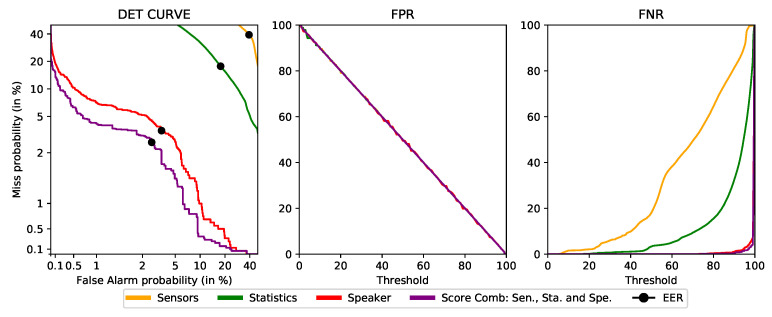
DET Curve, FPR and FNR curves after the score calibration.

The result of applying this transformation can be seen in the Figure 11, where the previous results are not affected and produces a higher resolution. After performing this calibration of scores, the setting FPR implied directly the selected threshold, thr=100−FPR. Now, after the calibration, all vectors and models share the same threshold.

Finally, to evaluate the proof-of-concept performance, the threshold is set to ninety, that is, where all models achieve a maximum FPR of 10%. In this case, the performance of the system is listed in the Table 11 for different metrics, FNR, Precision, Accuracy (ACC), and F1. All these different metrics are exposed because each one shows a characteristic from the models performance. At that threshold, it stands out how the FNR varies from 81% for the sensors to 1% for the speaker, and even if all scores are available, sensors, statistics, and speaker, with the score combination can be reduced to 0.34%. The Precision is low in all cases, always less than 30%, and even for sensors, it obtains a value of 6.97%. However, with respect to Accuracy all showed high values close to 90%, with very little resolution between them. The metric F1 does not exceed in any case more than 46%.

This shows that when only sensors vectors are present, the system has rather high rate of false negative alarms. This is mitigated quite dramatically when statistics are also available. Finally, when voice data are available, the system performance is really boosted. Besides, if all three data sets are available at the same time, the performance is even better.

It is true that the Sensors and Statistics are the data that can be accessed on a regular and high frequency, something desirable in a continuous authentication system, but the final decision of these systems is not necessarily based on the instant score of models accessed at high frequencies, but as confidence scores or authentication levels that takes into account time windows of different evidences and events. These results can help to calibrate these authentication level calculations, showing the performance of each kind of data and event and suggesting, for example, that such authentication levels would need to take into account one of more results of past statistics data scores and should not rely on sensors only.

Nevertheless, the most remarkable result is that voice clearly represents a highly accurate addition to authentication data, and that the events that allow for such information to be used have a great impact on the confidence scores provided by the system. When voice is present false negative rates become nearly negligible.

## 5. Conclusions

This work has designed, prototyped and validated the S3 platform, an AI-enabled continuous authentication system that uses data collected from smartphone motion sensors, applications usage statistics and speaker data. The proposed platform consists of a smartphone application responsible for collecting the data, and a framework, hosted in the cloud, where the data are processed for user authentication.

After the design, the prototype has been developed and a novel database has been collected to validate the framework. This dataset was collected using the application for 21 volunteers. The application monitored sensors and statics of application usage of the user without restriction during more than 60 days. The collection of the audio was done following a set protocol and reading a fixed text, to avoid collecting sensitive information and to respect user privacy. In this way, a novel database is publicly available in [9].

Once the prototypes were developed, a series of experiments were carried out. These experiments followed an anomaly detection approach, where the users’ profiles are extracted using unsupervised learning algorithms using only the data from individual users. That is, the algorithms only use training data from the user they model, and not any data from the rest of the known users of the system. Although the network used to extract the x-vector and the PLDA are supervised techniques, the proposed platform does not train them with its own data, but uses publicly available pre-trained models, and therefore maintains its non supervised learning approach.

First, it has been explored how informative each of the chosen dimensions is. The results showed that voice is the most solid, but it is also the least present in the use of the device, and it is also the times at which voice will be available are unforeseeable. Secondly, the aggregation of dimensions was tested in two ways, a vectors aggregation to build a vector with more attributes, and a score combination, where the score of the individual models are combined. The score combination achieved a little improvement over the best individual models, and both score combination and individual are better than vectors aggregation.

The third experiment evaluated how the size of the time window used for the training of the models affects the performance. It showed that 14 days is a good time-window to obtain good models, but with 5 days it starts to produce models with performance close to the ones trained with 14 days of collected data.

Finally, the performance of the platform was measured. It has been seen how the system, using a demanding 10% maximum FPR, improves its results dramatically if voice is added. The results are promising, but there is still a lot of room and need to improve them for real environments. When the voice is not present, which may happen frequently, the FNR rates are too high, but it has to be taken into account that, in this proof-of-concept, the techniques used were unsupervised and were not fully optimized, so it is expected that in future works their performance may be substantially improved.

## 6. Future Works

Future work will extend the experimentation with different algorithms and optimization of their parameters to achieve better results, especially with data from sensors and statistics. In addition to exploring other algorithms, it would be interesting to check other approaches to the problem, for example, to pose it as a classification problem and to use supervised machine learning techniques. The same approach used here to the labeling of the test set for evaluation purposes can also be used in order to obtain labeled data for training, and consider other users as examples of potential impostors. Although other users may not be the perfect mimic of the behavior of potential impostors (an impostor would supposedly try to emulate or imitate the original user), it is theorized that their data would help guide the machine learning algorithms. This would lead supervised training towards results better than those obtained by characterizing a class with no contrast behavior at all, as in the case of unsupervised learning techniques.

It is also a preferential line of work to focus on being able to obtain competitive single models that use aggregated data of different dimensions, and add other characteristics such as the time elapsed between different data. The use of supervised deep learning techniques may overcome the weaknesses shown in the experimentation of this work for these large size vectors.

The implementation of the parts of the S3 continuous authentication framework to be deployed in the smartphone is still pending, at least the modules and components necessary to calculate the authentication level. The model generation will still be hosted on the server because it is the part that consumes the most computing resources, and because it does not need to be done in real-time. Once this implementation is complete, the limitation of the current system of not providing authentication scores when the connection is interrupted will be solved, making the S3 platform independent of the internet, WiFi, or mobile data availability.

Once the platform design has been consolidated and it has sufficient precision to guarantee the users’ privacy, the next step is to investigate the possible attacks that the system may suffer. That is why our road map has the objective of studying and analyzing the effects of adversarial machine learning, and to explore possible countermeasures to guarantee users’ security and privacy.

Finally, it is of great interest to expand the database. Just 21 users is a rather small number but, due to the pandemic, it was the final number of volunteers recruited that provided full data at the end of the data collection process. When the situation returns to normal, it is intended to recruit more volunteers and thus increase the number of users’ data available for the scientific community to explore and experiment with.

## Figures and Tables

**Table 1:** Comparison of works dealing with continuous authentication.

Reference	Year	Dimension	Algorithm	Dataset	Results
[13]	2020	Sensors	Two-Stream CNN + PCA+ One-class SVM	Private Dataset (PD) with 100 users & Brain Run	4.57% EER, 4.65% FAR and 4.48% FRR 5.71% EER, 5.87% FAR and 5.56% FRR
[3]	2018	App’s usage statistics & sensors	IF, One-class SVM, Local Outlier Factor	PD with 2 users and 5 attackers	Precision: 77% Recall: 92% Accuracy: 82.5%
[14]	2020	Touch Screen & sensors	One-class SVM	PD with 30 users while they use the bank’s app	79% correct user identification, 13% false acceptance and 11.5% EER
[15]	2019	Touch Screen & Sensors	Three-layer deep network	HMOG dataset	88% accuracy and 15% EER values considering binary classification
[4]	2015	Touch Screen & voice & sensors	Not indicated	PD with 11 users	Approx 95% and 85% in security and usability respectfully
[5]	2011	Location & voice & multitouch & sensors	Not Indicated	PD with 4 users	The authors have evaluated the capabilities of three individual modalities
[8]	2013	Key Stroke & voice	KNN	User simulated	Achieve a 20% of EER for voice and 10% for key stroke. The authors indicate a 67% of reduction in required authentication
[16]	2020	Seven sources	SVM with RBF kernel	UMDAA-02	Accuracy ranging from 82.2% to 97.1%
[17]	2018	Sensors & keystrocke	one-class MPL	PD with 97 user	DialerAuth authenticate the user when he/she tap a phone number. The system shows a TAR of 85.77%
[18]	2019	Sensors & touch screen	Random Forest	PD with 85 user	AnswerAuth authenticate the user when he/she slide the button to answer a call and move the phone to the ear. The system show a TAR of 99.35%
[19]	2021	Sensors & statistics	XGBoost	AuthCode	The multi-device system achieved a 59.65% and 89.35% improvement in the FPR for mobile applications and mobile sensors respectively
[20]	2020	Gait	SVM	PD with 10 male user	The authors get the gait data from a wearable. The system achieved a EER of 0.01% and 0.16% with smart socks and smart shoes respectively
[10]	2021	Survey			It includes a collection of methodologies, feature extraction techniques, and relevant countermeasures against adversarial attacks.
[11]	2020	Survey			The authors present the challenges for each of the different biometric methods, highlighting potential future work and common gaps within the literature.
[12]	2020	Survey			The authors present an overview of continuous authentication methods in the IoT environment.

**Table 2:** Resume of databases for continuous authentication.

Reference	Name	Year	Dimension	Device	Details
[21]	HMOG	2015	Touch & sensor & keypress	Smartphone	100 volunteers in three scenarios: reading, writing, and navigating on a map
[23]	UMDAA-02	2016	Sensors & front camera & touchscreen& GPS & WiFi & Bluetooth	Smartphone	48 volunteers Not-Available
[25]	BrainRun	2019	Touch screen & sensors	Smartphone	2218 users playing the BrainRun game
[26]	SU-AIS BB-MAS	2019	Keystroke & sensors & touch screen	Desktop, smartphone, and tablet	117 participants in different scenarios
[27]	AuthCode	2020	Sensors & statistics	Laptop and smartphone	5 user for 60 days

**Table 3:** Comparison of works dealing with speaker recognition.

Reference	Year	Algorithm	Dataset	Results
[28]	2011	DNN	TIMIT & KING	A speaker representation is extracted from a DNN, obtaining intrinsic speaker-specific characteristics and generally outperform MFCCs
[29]	2014	DNN	Switchboard	The authors use a DNN to extract the Baum-Welch statistics for i-vector system.
[30]	2015	DNN	Switchboard	A single DNN for Speaker Recognition and Language Recognition task
[31]	2018	TDNN + PLDA	SITW & SRE16	State-of-the-art for Speaker Recognition. This technique improve the previous, the “i-vectors”. Their results are better by 44% in EER than the previous State-of-the-art
[33]	2020	TDNN-LSTM Attention NN	SRE19 CTS	The system achieves a 6.02% EER
[34]	2020	LSTM & TDNN & FTDNN	SRE19 CTS	A combination of the three system achieve a 2.59%
[35]	2020	TDNN + AM Centroid Loss	Librispeech	The authors show that the loss proposed outperforms other losses in performance and has a faster convergence
[36]	2021	TDNN + BGM	SITW & Voxceleb	The author replace the PLDA by a Bayesian Generative model, achieving a 3.142% EER for SITW
[37]	2020	2D-CNN	SITW & SRE16	System developed for wideband and narrowband, it achieves a 3.18% EER in the SITW and 5.44% EER in SRE16
[38]	2021	RBM & Autoencoder & Siamese	VoxCeleb-1	The author shows a relative improvement of 53 % compared to a system using i-vectors and PLDA.

**Table 4:** Database information collected.

Characteristic	Number
Users	21
Sensors vectors	417.128
Statistics app’s usage vectors	151.034
Speaker vectors	2.720
|– Call recordings	629
|– Voice messages	2.091

**Table 5:** Confusion Matrix.

		Real Condition	
		**True User (Positive)**	**Other User (Negative)**
Predicted Condition	True User	True Positives (TP)	False Positives (FP)
	Other User	False Negatives (FN)	True Negatives (TN)

**Table 6:** Global metrics for the best algorithms using sensors and statistics vectors. The best algorithm is a KNN with one neighbor.

Best Model	AUC	EER	F1-Max
Sensors	67.8199%	39.1179%	11.4649%
Statistics	89.5129%	17.7054%	36.6262%

**Table 7:** Results with the speaker vectors. The numbers between parenthesis are for call recordings and voice notes individually.

Model	AUC (%)	EER (%)	F1-Max (%)
Cosine Score	97.56 (95.73–98.01)	8.73 (11.46–7.98)	69.26 (57.54–72.68)
PLDA	99.54 (99.76–99.55)	3.40 (1.60–3.36)	89.29 (93.81–89.37)
IF	95.07 (96.58–95.27)	12.56 (9.11–12.78)	55.40 (58.73–58.64)
Sensors	53.02 (48.86–53.99)	48.83 (51.98–47.68)	9.35 (8.72–9.79)
Statistics	86.68 (89.53–86.01)	20.14 (18.47–20.51)	31.70 (37.73–31.70)

**Table 8:** Results for the individual, combined and aggregation models of the sensors and statistics vectors.

	AUC	EER	F1-Max
Sensors vectors	71.38%	34.68%	15.64%
Statistics vectors	90.38%	16.77%	39.02%
Score combination	90.38%	16.77%	39.02%
Sen&Sta KNN neighbors = 1	89.84%	18.93%	47.15%

**Table 9:** Results in scenes with voice for the models trained with voice, Sen&Sta&Voi vectors, and score combination from individual models.

	AUC	EER	F1-Max
Voice (PLDA)	99.73	2.83	92.44
Score Combination	99.74	2.59	92.53
Sen&Sta&Spe	93.26	12.61	73.95

**Table 10:** Thresholds and FNR for S3 platform in the respective work points, 30% and 10% for FPR.

	Threshold @FPR = 30%	FNR @FPR = 30%	Threshold @FPR = 10%	FNR @FPR = 10%
Sensors	99.9831	49.41%	99.9868	81.28%
Statistics	99.9963	9.36%	99.9980	32.34%
Speaker	52.5689	0.06%	60.3876	1.05%
Score combination: sensors and statistics	99.9963	9.36%	99.9980	32.34%
Score combination: sensors, statistics and speaker	90.5200	0.06%	92.0968	0.34%

**Table 11:** Performance of the S3 Platform at threshold equal to 90 (it implies a FPR of 10%).

	FNR	Precision	ACC	F1
Sensors	81.28%	6.97%	87.25%	10.16%
Statistics	32.34%	22.80%	89.06%	34.10%
Speaker	1.05%	26.63%	90.56%	41.97%
Score combination: sensors and statistics	32.34%	22.80%	89.06%	34.10%
Score combination: sensors, statistics and speaker	0.34%	29.58%	90.38%	45.62%

## Data Availability

The data presented in this study are openly available in FigShare at 10.6084/m9.figshare.14410229.v2, reference number 14410229.
